# Glucose is a pH-Dependent Motor for Sperm Beat Frequency during Early Activation

**DOI:** 10.1371/journal.pone.0041030

**Published:** 2012-07-20

**Authors:** Nadja Mannowetz, Petra M. Wandernoth, Gunther Wennemuth

**Affiliations:** Department of Anatomy and Cell Biology, Saarland University, Homburg/Saar, Germany; University Hospital of Münster, Germany

## Abstract

To reach the egg in the ampulla, sperm have to travel along the female genital tract, thereby being dependent on external energy sources and substances to maintain and raise the flagellar beat. The vaginal fluid is rich in lactate, whereas in the uterine fluid glucose is the predominant substrate. This evokes changes in the lactate content of sperm as well as in the intracellular pH (pH_i_) since sperm possess lactate/proton co-transporters. It is well documented that glycolysis yields ATP and that HCO_3_− is a potent factor in the increase of beat frequency. We here show for the first time a pathway that connects both parts. We demonstrate a doubling of beat frequency in the mere presence of glucose. This effect can reversibly be blocked by 2-deoxy-D-glucose, dichloroacetate and aminooxyacetate, strongly suggesting that it requires both glycolysis and mitochondrial oxidation of glycolytic end products. We show that the glucose-mediated acceleration of flagellar beat and ATP production are hastened by a pH_i_ ≥7.1, whereas a pH_i_ ≤7.1 leaves both parameters unchanged. Since we observed a diminished rise in beat frequency in the presence of specific inhibitors against carbonic anhydrases, soluble adenylyl cyclase and protein kinase, we suggest that the glucose-mediated effect is linked to CO_2_ hydration and thus the production of HCO_3_− by intracellular CA isoforms. In summary, we propose that, in sperm, glycolysis is an additional pH_i_-dependent way to produce HCO_3_−_,_ thus enhancing sperm beat frequency and contributing to fertility.

## Introduction

**Figure 1:**
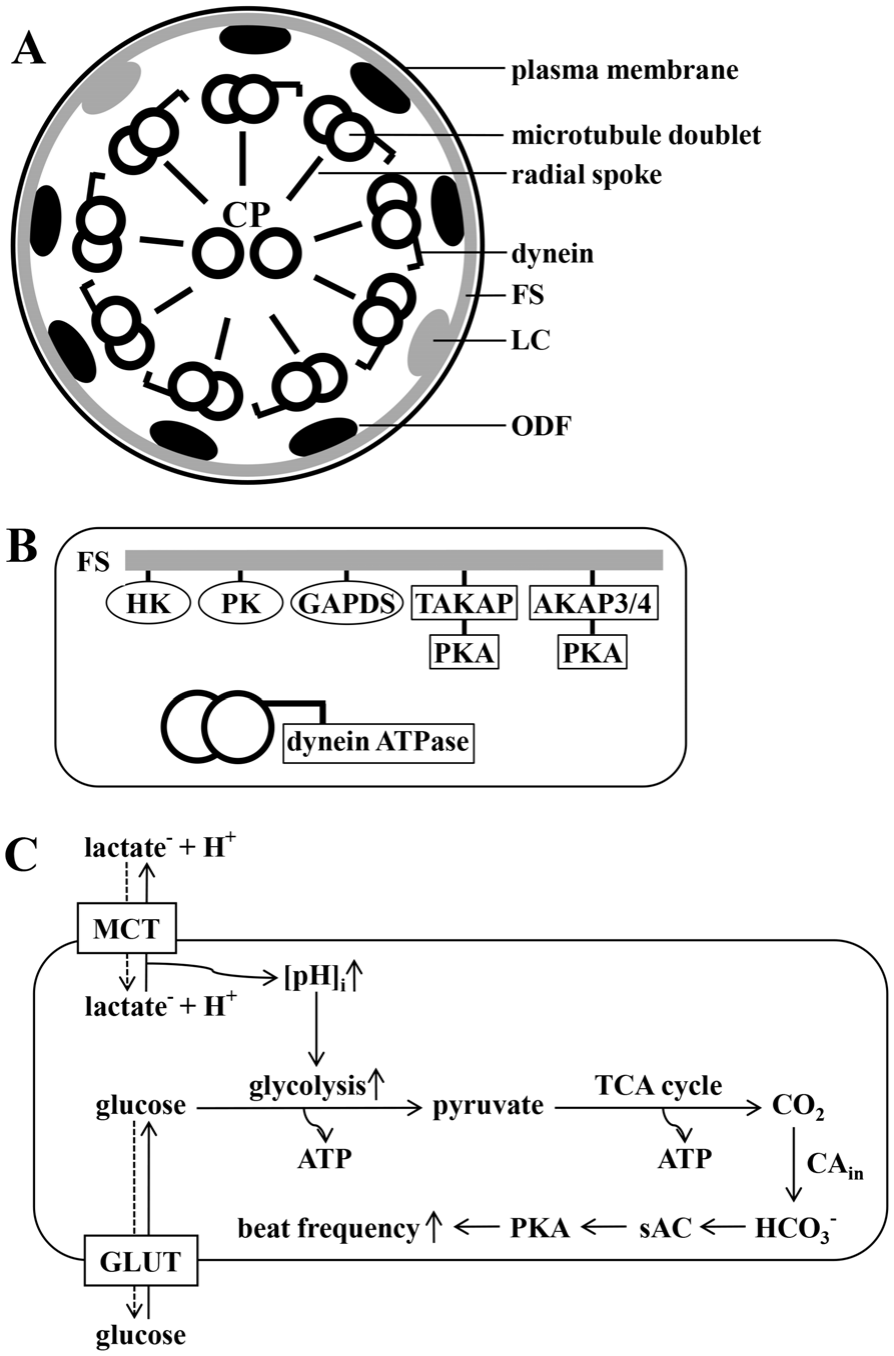
Organization of the principle piece of the sperm tail and proposed model for the interplay between pH_i_, glycolysis and production of HCO_3_−. **A**, Shown is a scheme of a cross section through the principle piece. Nine microtubule doublets - each carrying dynein arms - are connected via radial spokes to the central pair (CP), constituting the classical 9×2+2 core structure of the ciliar axoneme. Each microtubule doublet, in turn, is connected to two main longitudinal cytoskeletal structures – doublets 3 and 8 are fastened to the so-called longitudinal columns (LC), whereas doublets 1 and 2 and 4–7 are bound to the outer dense fibers (ODF). The outward facing area of each LC and ODF is tightly connected to the fibrous sheath (FS), a layer located right underneath the plasma membrane. **B**, Shown is an incomplete section of **A**, focusing on enzymes located to the FS, in close proximity to dynein ATPase. Glycolytic enzymes are hexokinase (HK), phosphokinase (PK), sperm specific glyceraldehyde 3-phosphate dehydrogenase (GAPDS) and lactate dehydrogenase A (LDHA). Proteins involved in the cAMP/HCO_3_- metabolism are A-kinase anchoring protein 3 and 4 (AKAP3/4) and testis A-kinase anchoring protein (TAKAP) with binding sites for protein kinase A (PKA). **C**, This drawing illustrates our working hypothesis. Glucose enters spermatozoa via glucose transporters (GLUT). Once lactate leaves the cell together with protons via monocarboxylate transporters (MCT), pH_i_ rises and glycolysis proceeds intensified. GLUT and MCT transport is bidirectional and the solid arrows indicate the proposed route of transport. The glycolytic end product pyruvate is metabolized during the mitochondrial citrate cycle yielding CO_2_ which will be hydrated to HCO_3_− by intracellular CA. HCO_3_−, in turn, directly activates the sperm specific sAC thereby stimulating PKA and leading to an increase in sperm beat frequency.

Once deposited in the vaginal fluid, which is rich in lactic acid, sperm travel through the cervix to enter the uterus where they encounter a fluid which is poor in lactate but rich in glucose and other glycolysable substrates [1]. Besides mitochondrial respiration, glycolysis is a major pathway for ATP production in murine spermatozoa [2], [3], [4]. Glycolytic enzymes are located in the fibrous sheath of the sperm tail [5] and some of them exhibit sperm-specific properties, such as type1 hexokinase (HK1S) [6], pyruvate kinase (PKS) [7] and glyceraldehyde 3-phosphate dehydrogenase (GAPDS) [8]. The fibrous sheath covers the outer dense fibers which, in turn, are located in close proximity to the microtubules. This arrangement permits the ATPases located on the dynein arms of the microtubule doublets (Fig. 1 A and B) to directly utilize the ATP generated during glycolysis to maintain bending of the sperm tail [5]. For mouse spermatozoa, muscle cells, leukocytes, erythrocytes and several other cell types it has been demonstrated that glycolytic key enzymes exhibit pH-dependency [6], [9], [10], [11]. We have previously shown that sperm possesses monocarboxylate/H+ co-transporters whose activity leads to intracellular acidification or alkalinization during application or removal of lactate and pyruvate [2]. With this work, we now investigate the way in which glycolysis is modulated in sperm by intracellular pH (pH_i_). Whereas the ATP generated during glycolysis helps sperm to maintain the resting beat stable, HCO_3_− is the only substance known so far in sperm to speed up their flagellar beat [10], [11], enabling them to travel along the uterus to reach the egg in the ampulla. Once inside the sperm cell, HCO_3_− directly activates the sperm-specific adenylyl cyclase (sAC) [12], [13] which, in turn, raises the intracellular level of cAMP, thereby stimulating protein kinase A (PKA) [14], [15], [16] and initiating the acceleration of beat frequency. Besides the above mentioned glycolytic enzymes the fibrous sheath is also a scaffold for A-kinase anchor protein 3 (AKAP3), A-kinase anchor protein 4 (AKAP4) and testis-specific A-kinase anchor protein 80 (TAKAP-80) (Fig. 1B), all of which contain binding sites for cAMP-dependent protein kinases [17]. Furthermore, AKAP3 of bovine sperm was shown to possess binding sites for phosphodiesterase 4A [18]. The near proximity of enzymes involved in both glycolysis and cAMP/HCO_3_− metabolism suggests that they together closely regulate sperm motility and beat frequency. HCO_3_− is available in the uterine fluid [19] and can enter sperm either directly by anion transporters as suggested by [20], [21], [22] or indirectly via carbonic anhydrases (CAs) [23], [24]. CAs catalyze the reversible hydration of CO_2_ to HCO_3_− and the majority of CA isoforms already identified is either membrane-bound proteins or existent in the cytoplasm. This raises the possibility that at least some portion of CO_2_ being produced during mitochondrial respiration is converted to HCO_3_−. With this work, we show evidence that glycolysis and cell respiration are interconnected for the production of HCO_3_− via CO_2_ as an additional way for sperm to regulate flagellar beat frequency pH-dependently during early activation.

## Materials and Methods

### Ethics Statement

NMRI mice were sacrificed in accordance with the university guidelines for animal protection. Killing of animals was applied for and approved by the animal rights office of the Saarland University, Homburg, Germany (ID 18/08).

### Solutions and Inhibitors

All buffer ingredients were obtained from Sigma-Aldrich (St. Louis, MO, USA). Standard high saline buffer (HS; pH 7.4) contained (in mM): 135 NaCl, 5 KCl, 2 CaCl_2_, 1 MgCl_2_, 20 HEPES, 5 glucose, 10 DL-lactate and 10 pyruvate. HS buffer, which was devoid of glucose, lactate or pyruvate was supplemented with equimolar concentrations of sucrose to maintain osmolarity. For pH calibration we used high potassium buffers K6.0 (pH 6.0), K7.0 (pH 7.0) and K8.0 (pH 8.0) which contained (in mM): 135 KCl, 5 NaCl, 2 CaCl_2_, 1 MgCl_2_, 5 glucose, 10 lactic acid and 10 pyruvic acid, as well as 30 MES (K6.0), HEPES (K7.0) or TAPS (K8.0). K6.0, K7.0 and K8.0 further contained 10 µM Nigericin to equilibrate extra- and intracellular pH. Carbonic anhydrase (CA) inhibitors EZA and AZA (final concentration 0.1 mM), protein kinase A (PKA) inhibitor H89 (final concentration 7.5 µM) and soluble adenylyl cyclase (sAC) inhibitor KH7 (final concentration 12.5 µM) were applied by local perifusion together with glucose. Malate-aspartate shuttle inhibitor aminooxyacetate (AOA) and pyruvate dehydrogenase kinase inhibitor dichloroacetate (DCA) were both applied with a final concentration of 3 mM to spermatozoa 5 min prior to stimulation with glucose. The forerun of sAC inhibitor 2-hydroxy estradiol (2'OH-E, final concentration 50 µM) was 10 min. In general, all inhibitors used in this study are membrane permeable.

### Animals and Sperm Preparation

Male NMRI mice were kept in the Animal Facility of the Dept. of Experimental Surgery of Saarland University. Sexually mature animals (age 8–20 weeks) were killed by Isoflurane (Baxter, Unterschleißheim, Germany) asphyxiation and cervical dislocation and epididymal sperm were prepared as previously described [25]. In brief, excised and cleaned cauda epididymidis and vas deferens were washed, transferred to 1 ml high saline buffer (HS) and incised 8–10 times. The sample was incubated for 20 minutes at 37°C and 5% CO_2_ to allow sperm to swim out into the medium. After spinning for 3 minutes at 300 x g and after washing three times in buffer HS, sperm were re-suspended in 0.5 ml HS buffer with a final concentration of 1–2×107 cells/ml.

### Measurement of Sperm Beat Frequency and Waveform Analysis

For sperm beat frequency measurements, 10 µl of the sperm suspension were transferred to a FluorDish™ and allowed to attach to the bottom. After 3–4 minutes, 3 ml of buffer solution were added to the adhered sperm. The sperm beating pattern was recorded with an inverted microscope (Nikon Eclipse TE2000-U) with a 40×0.65 N.A. objective. Images for waveform analysis were taken with a MotionScope M3-mono fast speed camera (Imaging Solutions, Ehingen, Germany) and collected with the MotionStudio 64 software (Imaging Solutions, Ehingen, Germany) at 300 Hz from a 1200×400 pixel region of the camera chip. Single sperm were selected for analysis with ImageJ, version 1.41, (http://rsb.info.nih.gov/ij/) and MetaMorph, version 7.1, (Visitron, Puchheim, Germany) was used to adjust brightness and contrast. Flagellar beat frequency was analysed using semi-automated analysis software written in Igor Pro (Wavemetrics, Lake Oswego, OR, USA), as previously described [19], [25]. Data was evaluated with Sigma Plot 11.0 (Systat Software, Chicago, IL, USA) and results are presented as means±s.e.m.

### Dye Loading, Photometry, Calibration and pH_i_ Analyses

0.25 ml of the sperm suspension (2–5×106 cells/ml) were mixed 1∶1 with HS buffer containing Pluronic®-F127 and 2′,7′-bis-(2-carboxyethyl)-5-(and-6)-carboxyfluorescein, acetoxymethyl ester (BCECF-AM; Invitrogen, Karlsruhe, Germany) both with a final concentration of 0.1 µM. After incubation for 10 min in the dark at room temperature, cells were centrifuged (300×g, 3 min) and re-suspended in 1 ml HS buffer. Subsequently, 10–20 µl of the sperm suspension were placed into an Attofluor® cell chamber (Invitrogen) and allowed to attach to the glass bottom. After 4–5 min, approx. 3 ml of buffer solution were added to the adherent cells and the chamber was put onto the stage of a Nikon Eclipse TE2000-U microscope equipped with a monochromator (TILL Photonics, Munich, Germany). Buffer solutions were applied to single sperm by local perifusion. Cells were excited monochromatically with λ1 = 436 nm and λ2 = 488 nm every 4 s for 25 ms and fixed emission was recorded at 535 nm. Calibration of the F436/488 ratio signal was performed as earlier described [2]. Data was recorded with the Axoscope 9.2 software (Molecular Devices, Sunnyvale, CA, USA) and the mean values of each experiment were smoothed with Igor Pro (Wavemetrics, Lake Oswego, OR, USA) using the Savitzky-Golay filter (2nd order, 71 points). Smoothed data was analyzed with Sigma Plot 11.0 (Systat Software, Chicago, IL, USA).

### Measurement of the ATP Content

Sperm of eight animals (∼8×107 cells/ml) were collected and washed in HS buffer containing the energy substrates lactate, pyruvate and glucose. After the last centrifugation step, cells were incubated for 30 min in HS buffer which did not contain any of the three energy sources. Then, the sperm suspension was divided into five equal portions and cells were collected by centrifugation. After removal of the supernatant, the five cell pellets were re-suspended at t  = 0 min in 1 ml HS buffer supplemented with lactate, glucose, 2-deoxy-D-glucose (2-DG), lactate/methylamine (MA) or glucose/MA. At t  = 0, 5, 10, 20 and 30 min, the intracellular ATP content of 1.6×106 cells (100 µl) was determined with the ATPlite Kit following the manufacturer's protocol (Perkin Elmer, Waltham, MA, USA). Data was collected as duplicates on a Lumat LB 9507 luminometer (Berthold Technologies, Bad Wildbad, Germany) at 10-second intervals. Relative fluorescence units (RFU) were transformed into terms of µM ATP on the basis of a regression line made up with ATP standards of 0, 1.25, 2.5, 5 and 10 µM. Raw data was analyzed with Sigma Plot 11.0 (Systat Software) and are presented as means±s.e.m.

### Measurement of the NADH/NADPH Content

Sperm were collected as described above and washed in HS buffer containing 10 mM lactate. After washing, the sperm suspension was split into equal portions (2.7×107 /ml) and incubated for 30 min at room temperature in HS buffer devoid of energy substrates. Cells were spun down and re-suspended in 1.5 ml HS buffer respectively supplemented with 10 mM lactate, 5 mM glucose or 1.3 mM 2-deoxy-D-glucose (2-DG). After 20 min, cells were centrifuged and each pellet was lysed in 0.6 ml buffer containing 300 mM triethanolamine and 4 mM MgSO_4_. To mimic prior intracellular conditions, the lysis buffer also contained 10 mM lactate, 5 mM glucose or 1.3 mM 2-DG. Since we previously could show that sperm pH_i_ averages at ∼6.8 and at ∼7.1 in the presence or absence of external lactate, lysis buffer with lactate was adjusted to pH 6.8, whereas lysis buffer containing glucose or 2DG had a pH of 7.1. 0.1 ml cell lysate were mixed with 2.7 ml of the respective lysis buffer in a disposable 2.5 ml polystyrene cuvette (Sarstedt, Nümbrecht, Germany) and absorption was determined over 200 s at 340 nm on an Ultrospec 3000 photometer (GE Healthcare, Buckinghamshire, GB). Raw data were analyzed with Sigma Plot 11.0 (Systat Software) and are presented as means±s.e.m.

### Determination of Cell Viability and Statistics

Cell viability during beat frequency or pH measurements was determined by counting 200 cells during the respective experimental conditions and calculating the percentage of motile and immotile sperm. To assess cell viability during ATP or the NADH/NADPH measurements an aliquot of the cell suspension was transferred at the beginning and at the end of the experiment onto a coverslip and 200 cells were counted and categorized as described above. If not otherwise mentioned in the text, cell viability in terms of motility was at least 91%. For statistical evaluation experimental data was compared on the basis of a t-test using Sigmaplot 11.0 (Systat Software, Chicago, IL, USA).

## Results

### Glucose as Energy Substrate Increases Sperm Beat Frequency Dramatically

**Figure 2:**
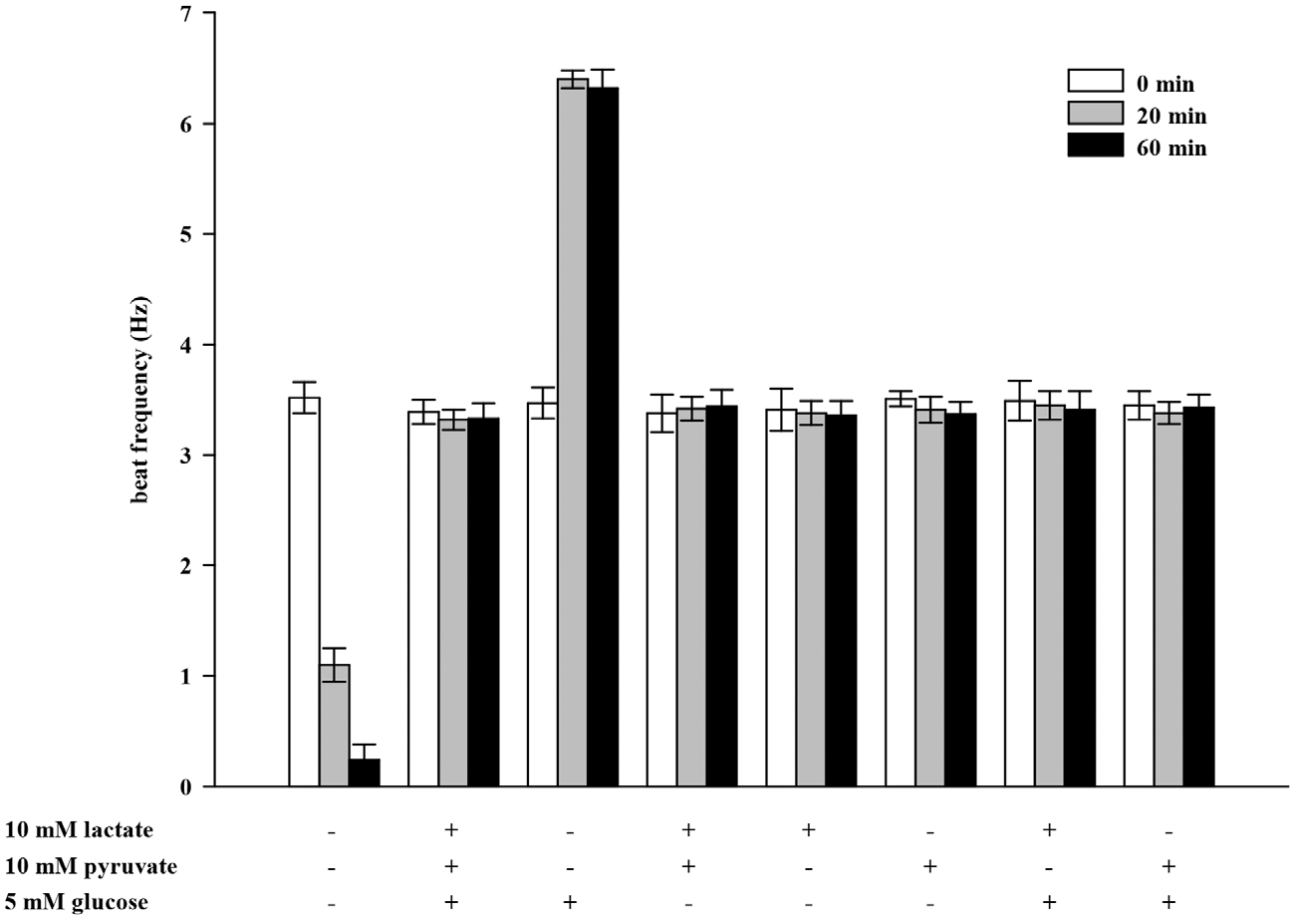
Glucose as energy substrate increases sperm beat frequency dramatically. Sperm were continuously perifused for 20 min with buffer HS containing different energy substrates (10 mM lactate, 10 mM pyruvate and 5 mM glucose) as indicated. Beat frequency of single sperm was analyzed at t  = 0 min (white bars), t  = 20 min (gray bars) and t  = 60 min (black bars). Shown are mean values±s.e. of 35 cells of 3 animals. With no energy substrate present, flagellar beat declines significantly compared to t  = 0 min (***p<0.001). With glucose as the mere substrate, beat acceleration is significant compared to t  = 20 min and 60 min (***p<0.001) of all other substrate compositions.

Sperm require an exogenous metabolic substrate to sustain prolonged beating of the flagellum. Lactate, pyruvate and glucose are energy substrates present in the female genital tract fluids. We therefore investigated sperm beat frequency at t  = 0, 20 and 60 min in response to these substrates which were applied by continuous perifusion (Fig. 2). After incubation in a medium without energy substrates, flagellar beat frequency decreases significantly from 3.52±0.14 Hz (0 min) to1.10±0.15 Hz (20 min) and 0.24±0.14 Hz (60 min) which corresponds to the increase in the percentage of immotile sperm from 6% to 94% (data not shown). With all three substrates present, initial beat frequency of 3.39±0.11 Hz remains unchanged after 20 min (3.32±0.09 Hz) and 60 min (3.33±0.14 Hz). This is also the case when lactate and pyruvate are added together (3.38±0.17 Hz vs. 3.42±0.11 Hz and 3.44±0.15 Hz). The flagellar beat does not alter in the presence of lactate (3.41±0.19 Hz vs. 3.38±0.11 Hz and 3.36±0.13 Hz) or pyruvate (3.51±0.07 Hz vs. 3.41±0.12 Hz and 3.37±0.11 Hz) applied alone, neither does co-application with glucose influence sperm beat frequency (3.49±0.18 Hz vs. 3.45±0.13 Hz and 3.41±0.17 Hz (lactate/glucose) and 3.45±0.13 Hz vs. 3.39±0.10 Hz (pyruvate/glucose)). When glucose is applied alone, beat frequency increases significantly more than 2-fold from 3.47±0.14 Hz (0 min) to (6.40±0.08 Hz (20 min) and 6.32±0.17 Hz (60 min). We sought explanations for the accelerating action of glucose and its suppression by lactate or pyruvate.

### 2-deoxy-D-glucose (2DG) Cannot Mimic the Effect of Glucose on Sperm Beat Frequency

**Figure 3:**
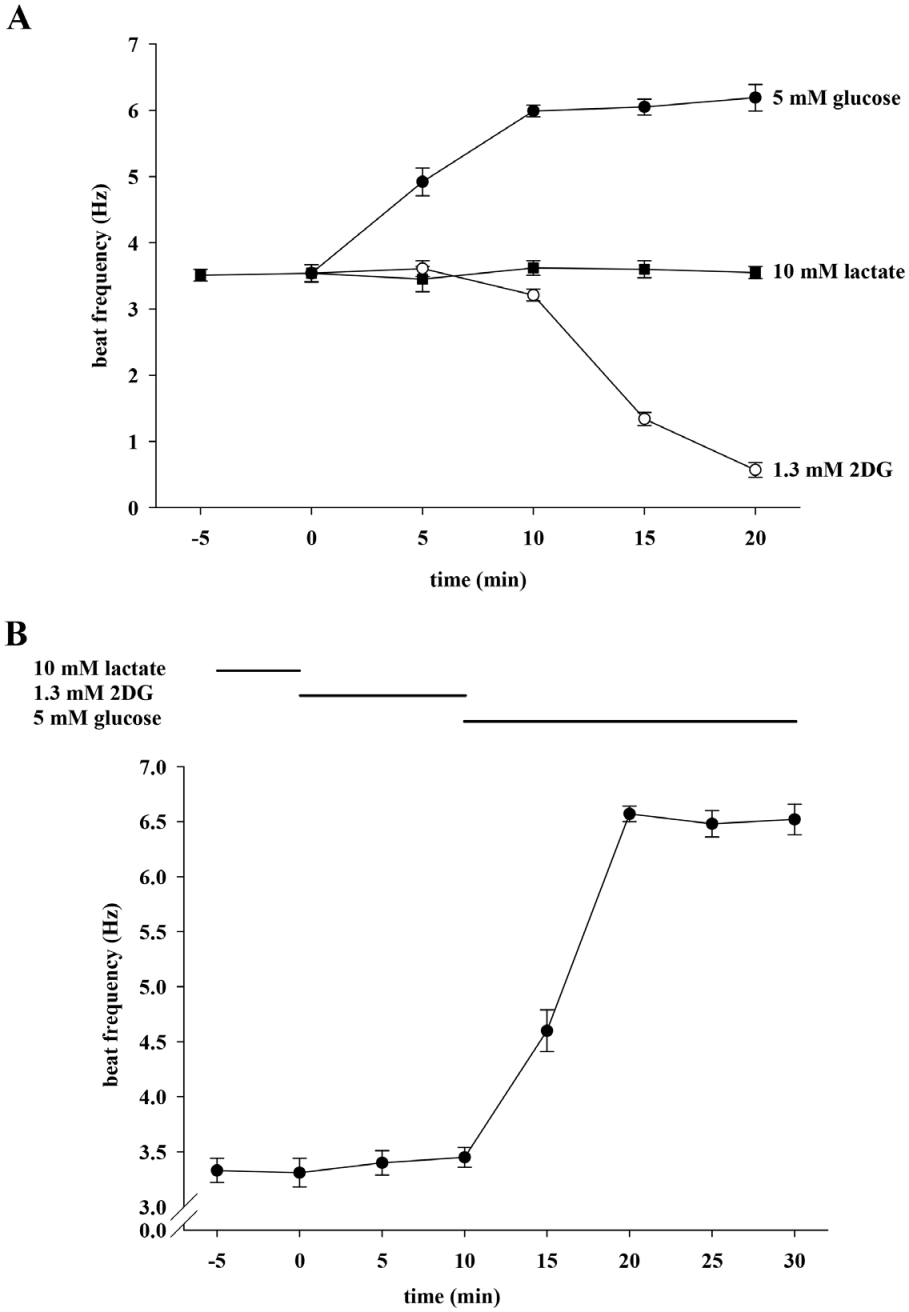
2-deoxy-D-glucose (2DG) cannot mimic the effect of glucose on sperm beat frequency. **A**, Sperm were initially perifused with buffer HS containing 10 mM lactate. During subsequent 20 min, perifusion was switched to buffer containing 5 mM glucose (filled circles) or 1.3 mM 2DG (open circles) as energy source. For control measurements, sperm remained in lactate-containing buffer (filled squares). Significant changes were analyzed relating to t  = 0. For clarity, statistical symbols are omitted in some of the following figures but explained in the respective figure legend. At t ≥5 min, values were significantly increased (***p<0.001) during application of glucose as well as at t ≥15 min during application of 2DG. **B**, Sperm were first perifused with buffer HS containing 10 mM lactate only. As indicated, sperm were then stimulated for 10 min with 1.3 mM 2DG followed by another 20-min treatment with 5 mM glucose. Significance was analyzed relating to t  = 0 min and values were significantly different at t ≥15 min (***p<0.001). Each plot represents mean values±s.e. of 36 cells of 3 animals.

We considered the possibility that the accelerating action of glucose is dependent on its metabolism by glycolysis. Figure 3A shows that, as in Figure 2, lactate maintains a constant basal beat frequency of 3.54±0.13 Hz for 20 min (3.55±0.09 Hz), whereas glucose increases the beat significantly to 6.19±0.20 Hz. The non-metabolizable analog 2DG is not able to sustain the basal beat frequency, and after 20 min cells display a significantly reduced beat of 0.57±0.11 Hz. Furthermore, the percentage of motile cells decreases from 93% to 38% (data not shown). Figure 3B illustrates that the 2DG action is not permanent since glucose accelerates the beat from 3.45±0.09 Hz to 6.52±0.11 Hz when applied after a 10 min treatment with 2DG. These results demonstrate that 2DG can be displaced by glucose and suggest that the accelerating action of glucose requires its processing by glycolysis. Although we measured the effects of different substrates on sperm beat frequency also in this and the following sets of experiments over at least 60 min we mainly show only shorter time points for the following reasons: (i) Frequency values did not change significantly over 60 min or longer and therefore presenting these data would not provide more information. (ii) Our main interest in this work is more in early sperm activation than in long term effects on sperm.

### Lactate and Pyruvate Reversibly Prevent the Enhancing Effect of Glucose on Sperm Beat Frequency

**Figure 4:**
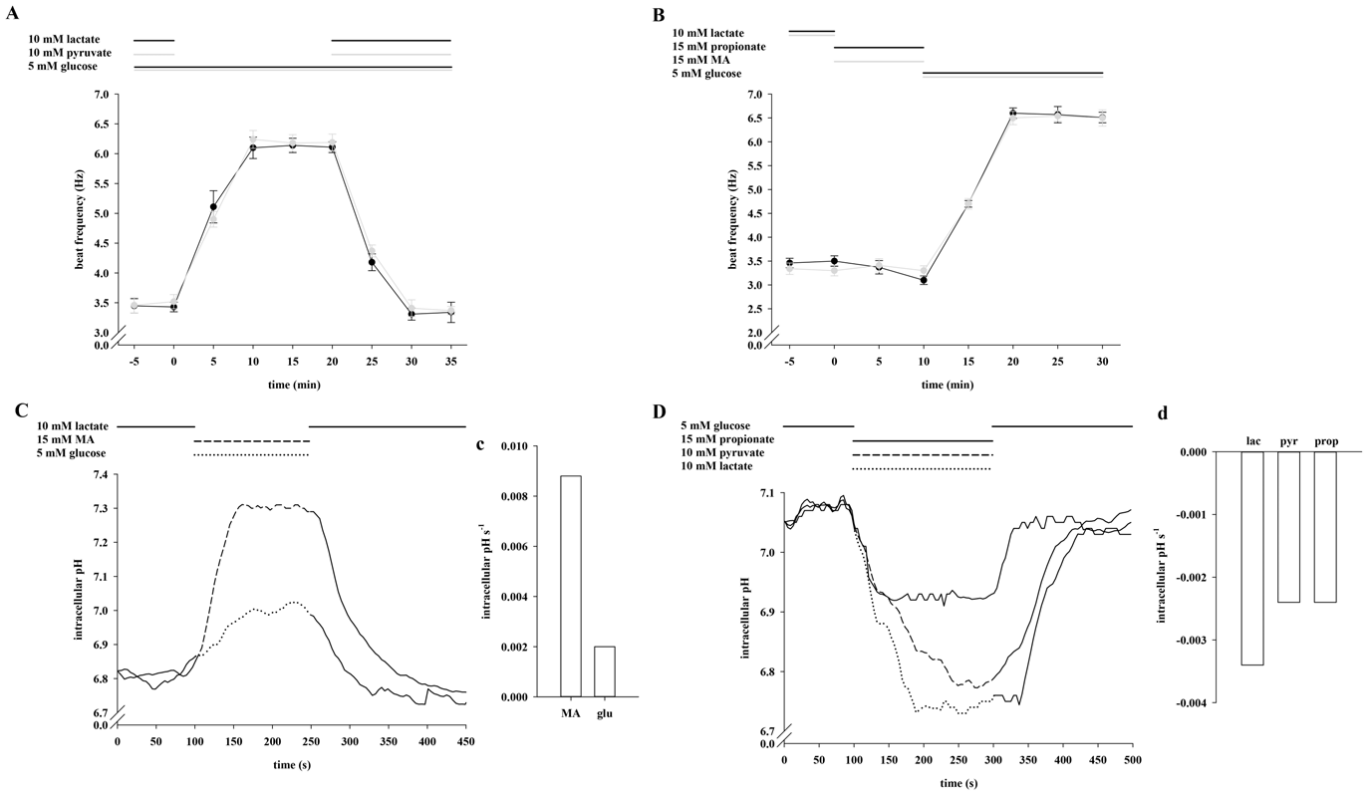
Lactate and pyruvate reversibly prevent the enhancing effect of glucose on sperm beat frequency. **A**, Sperm were exposed to 5 mM glucose for 40 min. During the first 5 min and the last 15 min, either 10 mM lactate (black plot) or 10 mM pyruvate (gray plot) were co-applicated by continuous perifusion. Significant changes were analyzed relating to t  = 0. At t  = 5, 10, 15, 20 and 25 min, values were significantly increased (***p<0.001) in both plots compared to t  = 0 min. **B**, Primarily, sperm were locally perifused with buffer HS containing 10 mM lactate. Then, stimulation occurred in the presence of 15 mM propionate (black plot) or 15 mM MA (gray plot) for 10 min, followed by another 20 min stimulation with 5 mM glucose. Compared to t  = 0 min, the values were significantly increased at t ≥15 min (***p<0.001) after removal of propionate or MA. The plots of **A** and **B** represent mean values±s.e. of 41 cells of 3 animals. **C**, Sperm were perifused for 100 s with buffer HS containing 10 mM lactate before being stimulated with 15 mM MA (dashed line) or 5 mM glucose (dotted line) for 150 s. After stimulation, cells were again bathed for 200 s in lactate-containing buffer solution. **D**, After a 100 s-treatment with 5 mM glucose, cells were stimulated for 200 s with 15 mM propionate (solid line), 10 mM pyruvate (dashed line) and 10 mM lactate (dotted line) before perifusion was switched back to lactate-containing buffer for another 200 s. The plots of **C** and **D** represent mean values of 25–33 cells of 3 animals. Mean slopes in **c** and **d** were analyzed during the first 50 s during application of the different stimulatory substrates and are presented as intracellular pH s-1.

We next evaluated whether the lack of frequency acceleration with glucose in combination with lactate or pyruvate is also reversible (Fig. 4A). After a 5 min exposure to either lactate/glucose or pyruvate/glucose, a 20 min application of only glucose significantly increases sperm beat frequency from 3.43±0.08 Hz to 6.10±0.18 Hz (lactate/glucose) and from 3.52±0.12 Hz to 6.24±0.15 Hz (pyruvate/glucose) respectively. Beat frequency declines to initial values during subsequent re-application of lactate (3.31±0.10 Hz) and pyruvate (3.41±0.14 Hz). Since we have previously shown that monocarboxylate/proton co-transporters are functionally active in sperm, we monitored pH_i_ shifts during application of lactate, pyruvate and glucose. To illustrate alkaline shifts, sperm were initially stimulated with lactate (Fig. 4C), whereas cells were first incubated in buffer supplemented with glucose to measure acidic shifts (Fig. 4D). Figure 4C illustrates that the basal mean pH_i_ of 6.81 in the presence of lactate rises to 7.05 within 70 s during application of glucose and reaches initial levels during recovery in lactate-containing buffer. We additionally determined the slopes during the first 50 s of glucose application resulting in a rise rate of 2.0 e-3 pH_i_ s-1 (Fig. 4c). In glucose containing buffer, sperm exhibit a resting pH_i_ of 7.09 which declines within 100 s to 6.67 (lactate) and within 150 s to 6.77 (pyruvate) and increases to initial values upon reversion to glucose-containing buffer (Fig. 4D). The slopes (Fig. 4d) average at −3.4 e-3 pH_i_ s-1 (lactate) and −2.4 e-3 pH_i_ s-1 (pyruvate). Since both the proton and the lactate anion gradient are driving forces for MCT activity, we also determined sperm frequency in HS buffer containing 5 mM glucose as usual but less lactate and less protons (5 mM lactate, pH of 8.0) (Fig. S1). This led to a significant increase of the beat from 3.50±0.07 Hz to 4.82±0.23 Hz. Due to pH_i_ shifts during the change of external lactate/pyruvate content we asked if the glycolysis-dependent acceleration could be mimicked solely by shifts in pH_i_. (Fig. 4B). We therefore exposed sperm to the weak base methylamine (MA) and the weak acid propionate, both of which can be taken up but are not metabolized by the cells. Application of MA leads to an increase of pH_i_ from to 6.81 to 7.31 within 70 s (Fig. 4C) with a slope of 8.8 e-3 pH_i_ s-1 (Fig. 4c). Propionate induces an acidic shift from 7.09 to 6.92 within 50 s (Fig. 4D) with a decrease rate of −2.4 e-3 pH_i_ s-1 (Fig. 4d). However, no significant changes in basal beat frequency occur during 10 min exposure to either MA (3.30±0.11 Hz vs. 3.32±0.10 Hz) or propionate (3.50±0.11 Hz vs. 3.10±0.09 Hz). During the following 20 min application of glucose sperm beat frequency significantly increases to 6.60±0.11 Hz (after propionate) and to 6.50±0.14 Hz (after MA) respectively. From these results we conclude not only that the accelerating action requires glucose but also that this effect cannot be explained by sole changes in pH_i_.

### The Enhancing Effect of Glucose on Sperm Beat Frequency is Hastened by Methylamine (MA) and Inhibited by Propionic Acid

**Figure 5:**
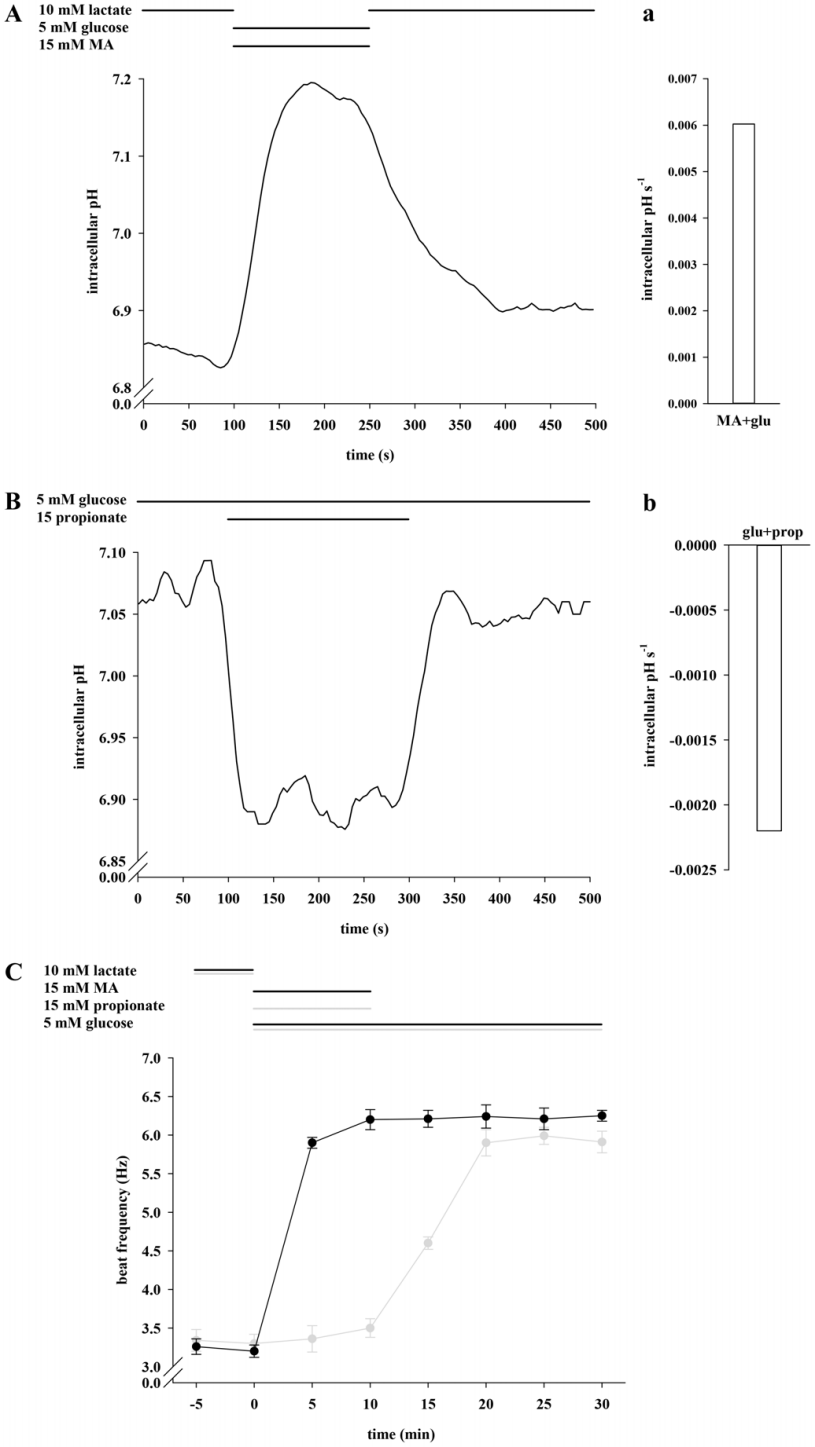
Methylamine (MA) hastens and propionic acid inhibits the enhancing effect of glucose on sperm beat frequency. **A**, Sperm were exposed for 100 s to 10 mM lactate before being stimulated for 150 s with 5 mM glucose and 15 mM MA. Subsequently, cells were allowed to recover for 200 s in lactate-containing buffer. **B**, Cells were treated for 500 s with 5 mM glucose of which propionate was additionally present during 200 s as indicated. The plots of **A** and **B** show mean values of 25–33 cells of 3 animals. Mean slopes (in **a** and **b**) were analyzed as described in the previous figure legend. **C**, After primary perifusion with 10 mM lactate, sperm were perifused for 30 min with buffer HS containing 5 mM glucose of which 15 mM MA (black plot) or 15 mM propionate (gray plot) were concurrently present first 10 min (t  = 0 min to t = 10 min). Significant changes were analyzed relating to t  = 0 min and apparent at t ≥5 min (co-application of MA) and at t ≥15 min (after removal propionate) with ***p<0.001. Shown are mean values±s.e. of 40 cells of 3 animals.

Since glycolytic key enzymes show pH interconnection, we examined whether the glycolysis-dependent accelerating action of glucose could be modulated by changes in pH_i_ during co-application of glucose and MA or glucose and propionate. Figure 5A illustrates that the pH_i_ rises within 100 s from 6.85 to 7.18 by co-application of glucose and MA. The rise rate averages at 6.0 e-3 pH_i_ s-1 (Fig. 5a). Co-application of glucose and propionate leads to a pH_i_ shift from 7.09 to 6.90 within 25 s (Fig. 5B) with a decline rate of −2.2 e-3 pH_i_ s-1 (Fig. 5b). After a 5 min exposure to lactate, co-application of glucose and MA for 10 min leads to a significant acceleration of sperm beat frequency within the first 5 min (3.20±0.08 Hz vs. 5.90±0.07 Hz; Fig. 5C). The 5-min value is significantly higher than that of the control measurements performed with glucose (4.47±0.18 Hz; data not shown). Conversely, co-application of glucose and propionate does not change the beat within 10 min (3.30±0.12 Hz vs. 3.50±0.12 Hz). After the subsequent application of glucose, sperm accelerate to 6.30±0.15 Hz (after MA + glucose) and 5.90±0.17 Hz (after propionate + glucose). We interpret these results as suggesting that changes in pH_i_ directly alter the rates of glycolysis and thus the glycolysis-dependent acceleration of the flagellar beat.

### The Glucose-mediated Acceleration of Sperm Beat Frequency Involves Mitochondria and can be Mimicked by Succinate

**Figure 6:**
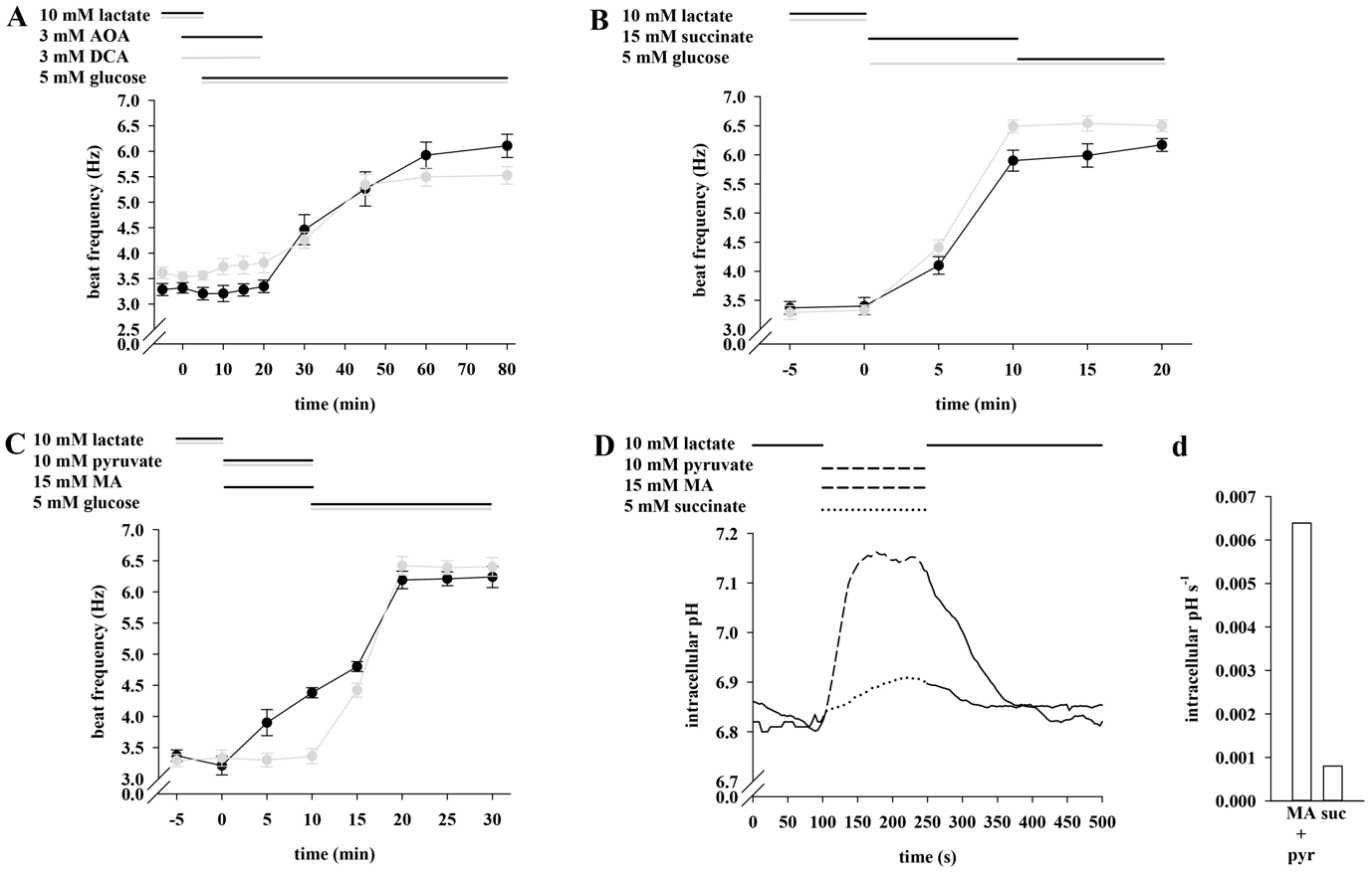
The glucose-mediated acceleration of sperm beat frequency involves mitochondria and can be mimicked by succinate. **A**, After a 5 min pre-run of either 3 mM AOA (black plot) or 3 mM DCA (grey plot) in buffer HS containing 10 mM lactate, sperm were perifused with the respective inhibitor in the presence of 5 mM glucose for 15 min. AOA/DCA were washed out for a subsequent period of 60 min with HS buffer supplemented with 5 mM glucose. Compared to t  = 0 min, the frequency values significantly increased at t ≥30 min (***p<0.001). **B**, After initial perifusion with 10 mM lactate, sperm were first stimulated for 10 min with 5 mM succinate and then for another 10 min with 5 mM glucose (black plot). For control measurements (gray plot), sperm were stimulated for 20 min with only glucose. Compared to t  = 0 min, the frequency values significantly increased at t  = 5 min (**p<0.05) and at t ≥10 min (***p<0.001) in both approaches. **C**, Treatment with 10 mM lactate was followed by a 10-min perifusion with 10 mM pyruvate and 15 mM MA simultaneously present in the buffer and a subsequent stimulation with 5 mM glucose for further 20 min (black plot). During the control measurements (gray plot), sperm were treated the same way except for omitting MA. Co-application lead to a significant increase of flagellar beat at t  = 5 min with **p<0.05 and at t ≥10 min with ***p<0.001. In the control significance of ***p<0.001 was reaches at t ≥15 min. Shown are mean values±s.e. of 38 cells of 3 animals. **D**, Sperm were exposed for 100 s to 10 mM lactate before being stimulated for 150 s with 10 mM pyruvate +15 mM MA (dashed line) or 5 mM succinate (dotted line). Thereafter, cells were allowed to recover for 200 s to initial values. The plots of **A** to **D** represent mean values±s.e. of 25–33 cells of 3 animals. The graphs in **d** show mean values of 30–35 cells of 3 animals and mean slopes were analyzed as described in the penultimate figure legend.

Since glycolytic end products are further metabolized by oxidate phosphorylation, we asked if blocking mitochondrial processes in the presence of external glucose could prevent an increase in flagellar beat. To address this question, sperm beat frequency was determined in the concomitant presence of glucose and inhibitors against both the malate-aspartate shuttle (aminooxyacetate, AOA) and the pyruvate dehydrogenase kinase (dichloroacetate, DCA). Both inhibitors were applied in a concentration of 3 mM, which did not affect flagellar beat during 30 min (data not shown). Figure 6A shows the effect of AOA and DCA in combination with glucose on sperm beat frequency. After perifusing the cells with inhibitors and glucose for 15 min, flagellar beat averages at 3.35±0.12 Hz (AOA) and 3.81±0.19 Hz (DCA), which is nearly identical to the resting beat of 3.32±0.10 Hz (AOA) and 3.53±0.10 Hz (DCA). During removal of the inhibitors beat frequency increases to a maximum of 6.11±0.23 Hz (AOA) and 5.53±0.17 Hz (DCA) within 60 min. To further demonstrate the supportive effect of respiration-dependent processes on sperm beat frequency, we applied succinate as a substrate of the mitochondrial citric acid cycle. After a 5 min exposure to lactate, the application of the respiratory substrate succinate accelerates the basal beat of 3.40±0.15 Hz significantly to 5.90±0.18 Hz (Fig 6B). Subsequent application of glucose for 10 min produces no further significant effect (6.17±0.11 Hz). These values are similar to those of the control measurements during which sperm were exposed for 20 min to glucose, resulting in 3.33±0.09 Hz (0 min), 6.49±0.11 Hz (10 min) and 6.50±0.10 Hz (20 min). According to Figure 4D, application of pyruvate leads to an intracellular acidification of approx. 0.25 pH units and to a lack of accelerating effect on sperm beat frequency (Fig. 2 and 4A). Since MA hastens the glucose-mediated acceleration of flagellar beat, we investigated if a higher pH_i_ would also support a pyruvate-mediated increase in beat frequency. Figure 6C shows that sperm do not accelerate their resting beat during a 10 min application of pyruvate (3.33±0.13 Hz vs. 3.36±0.12 Hz), whereas, co-application of pyruvate and MA significantly increases beat frequency from 3.21±0.15 Hz to 4.38±0.08 Hz. Figure 6D illustrates that the pH_i_ remains nearly unchanged during the addition of succinate (6.81 vs. 6.89) but increases from 6.85 to 7.15 during co-application of pyruvate and MA. The corresponding rise rates (Fig. 6d) average at 8.0 e-4 pH_i_ s-1 (succinate) and 6.4 e-3 pH_i_ s-1 (pyruvate + MA). Taken together the results suggest that the glucose-mediated acceleration of sperm beat frequency involves mitochondrial metabolic processes and that succinate is as effective as glucose, whereas pyruvate is a potent accelerant only at a pH_i_ ≥7.05.

### Production of ATP and NADH/NADPH is Dependent on the Substrate and the pH_i_

**Figure 7:**
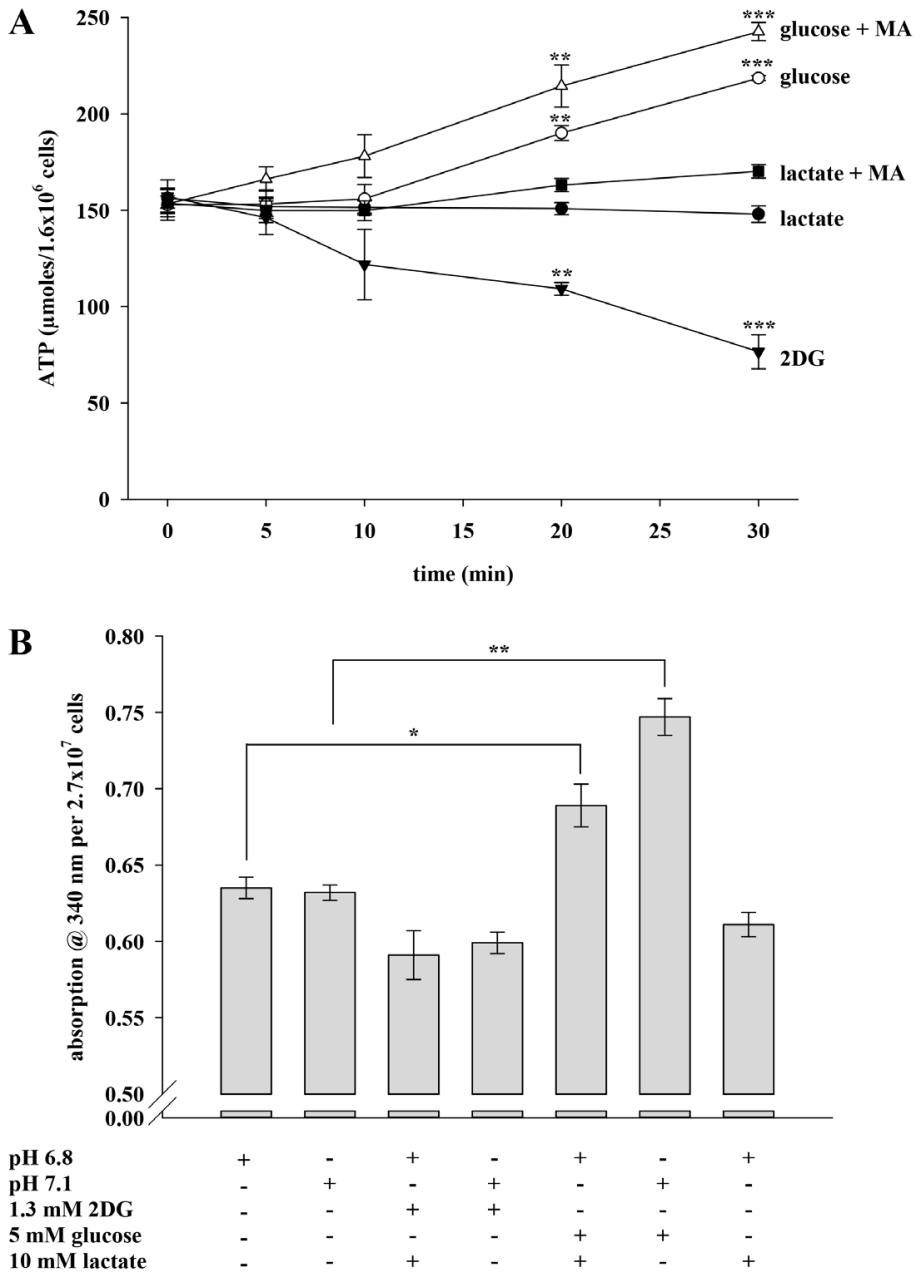
ATP and NDH/NADPH production is dependent on the substrate and the pH_i_. **A**, Sperm prepared as described in the Materials and Methods section were transferred to buffer HS which was supplemented with 1.3 mM 2DG (filled triangles), 10 mM lactate (filled circles), 10 mM lactate +15 mM MA (filled squares), 5 mM glucose (open circles) or 5 mM glucose +15 mM MA (open triangles). The ATP content was determined at t  = 0, 5, 10, 20 and 30 min. Each line represents mean values±s.e. of 4 independent experiments. Significant changes were analyzed relating to t  = 0 min of the respective plot with **p<0.05 and ***p<0.001. **B**, At t  = 0, sperm were starved out and directly homogenized in HS buffer with pH 6.8 or pH 7.1. After starvation, sperm were further incubated in buffer containing energy substrates as indicated before being homogenized. Absorption at 340 nm was measured over 200 s. Each bar represents mean values±s.e. of 2 independent experiments. *p<0.1 and ***p<0.001.

Glycolysis is a major mechanism for murine sperm to generate ATP. We reasoned that not only beat frequency but also the intracellular ATP content could be regulated by the pH_i_ since glycolytic key enzymes show pH dependency. Figure 7A compares the ATP content of sperm incubated with 2DG as well as lactate and glucose either in the simultaneous absence or presence of the weak base MA over a period of 30 min. As expected, application of 2DG is accompanied by a falling ATP content from 154.59±4.97 to 76.58±8.87 µmoles/1.6×106 cells. This is accompanied by a decrease in the percentage of motile cells from 94% to 33% (data not shown). Lactate which sustains the beat without acceleration also maintains a constant ATP content (154.59±4.97 vs. 147.97±4.26 µmoles/1.6×106 cells). Co-application of lactate and MA leads to slightly higher values (154.59±4.97 vs. 170.15±3.51 µmoles/1.6×106 cells). The accelerating effect of glucose is accompanied by a rise in sperm ATP. The ATP content remains constant during the first 10 min of glucose application, but then increases to 190.02±3.85 µmoles/1.6×106 cells (20 min) and 218.59±1.28 µmoles/1.6×106 cells (30 min). With the weak base MA concomitantly present, the ATP content increases from the beginning resulting in higher values at all time points (166.15±6.31, 178.07±11.11, 214.45±10.97 and 242.73±4.66 µmoles/1.6×106 cells). In addition to ATP, we also determined the amount of the reduction equivalents NADH/NAPDH in sperm lysates by measuring the absorption at 340 nm. Figure 7B illustrates that absorption values average at 0.635±0.007 and 0.632±0.005 when cells were incubated in HS buffer (pH 6.8 or 7.1) devoid of energy substrates. These results indicate that extracellular pH (pH_ex_) does not influence the generation of reduction equivalents. To determine whether pH_i_ has an effect on the content of NADH/NADPH, we next investigated cells which were incubated with 2DG or glucose either in the presence or absence of lactate to evoke a pH_i_ of 6.8 or 7.1. Sperm exposed to 2DG and lactate display a mean absorption value of 0.591±0.016, which is comparable to the values of sperm incubated with 2DG only (0.599±0.007). Feeding cells with glucose leads to absorption values of 0.747±0.012, whereas the concomitant presence of lactate causes a significant decrease to 0.689±0.014. In the presence of lactate only, mean absorption reaches 0.611±0.008. We interpret these results to indicate that the intracellular content of ATP and reduction equivalents may be regulated by both the available energy substrate and the pH_i_.

### The Enhancing Effect of Glucose on Sperm Beat Frequency can be Diminished with Specific Inhibitors against CAs, sAC and PKA

**Figure 8:**
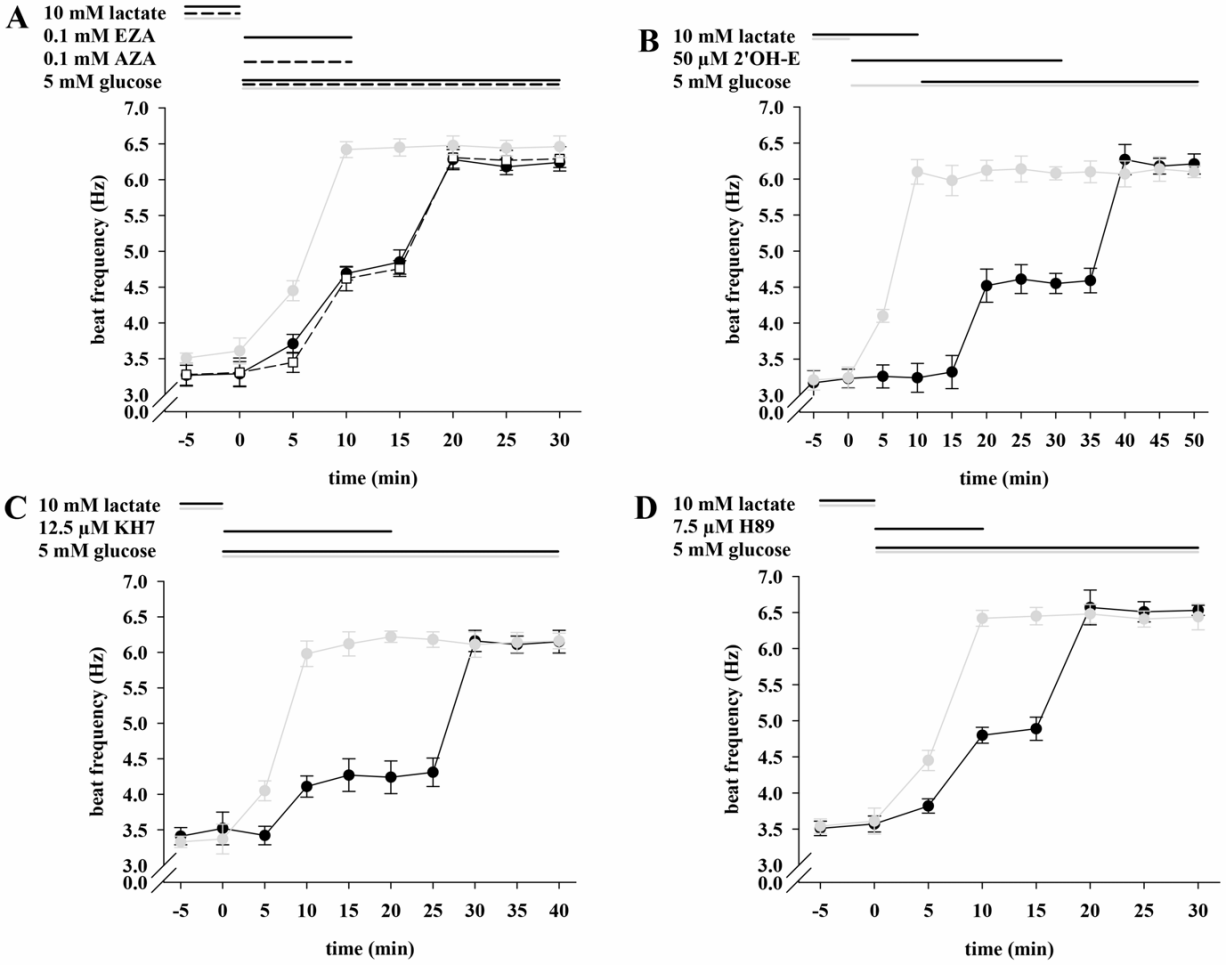
The enhancing effect of glucose on sperm beat frequency can be diminished with specific inhibitors against CAs, sAC and PKA. A, Sperm were initially perifused with 10 mM lactate. Then, perifusion was switched for 10 min to buffer HS either containing 0.1 mM EZA (solid line and filled circles) or AZA (dashed line and open squares) with the additional presence of 5 mM glucose. Subsequently, sperm were stimulated for 20 min with 5 mM glucose only. In the control measurements (gray plot), sperm were stimulated the same way except for omitting CA inhibitors. Significant changes were analyzed relating to t  = 0 min and accounted for ***p<0.001 at t ≥10 min under inhibiting and for ***p<0.001 at t ≥5 min in the control. B, Sperm were initially perifused with 10 mM lactate followed by application of 50 µM 2'OH-E for 10 min. For the next 20 min, sperm were stimulated with 50 µM 2'OH-E and 5 mM glucose. Subsequently, perifusion was switched for another 20 min to buffer HS containing 5 mM glucose only (black plot). Under inhibiting conditions, values were significantly increased (***p<0.001) at t ≥20 min and in the control at t ≥5 min. C, After perifusing with lactate, sperm were stimulated with 12.5 µM KH7 and 5 mM glucose for 20 min, and with 5 mM glucose only for another 20 min (black plot). In the respective control measurements (gray plots), sperm were treated the same way except for omitting sAC inhibitors. Significant changes were analyzed relating to t  = 0 min and averaged at **p<0.05 (t ≥10 min) and at ***p<0.001 (t ≥30 min) under inhibiting conditions. In the control, significance values of **p<0.05 at t  = 5 min and of ***p<0.001 at t ≥10 min were apparent. D, The same perifusion protocol was used as in A, whereupon 7.5 µM of the PKA inhibitor H89 was used instead of EZA and AZA (black plot). For control measurements (gray plot), sperm were perifused the same way except for omitting H89. Significant changes were analyzed relating to t  = 0 min of the respective plot with ***p<0.001 at t ≥10 min under inhibiting conditions and ***p<0.001 at t ≥10 min in the control. In each plot mean values±s.e. of 35 cells of 3 animals are shown.

Although intracellular ATP content increases significantly at a pH_i_ ≥7.1, this does not explain the acceleration of beat frequency since only HCO_3_− - but not ATP - is known to speed flagellar beat. We therefore considered whether the accelerating effects of glucose and succinate involve oxidative production of CO_2_ and CA-dependent cytosolic hydration to HCO_3_−. Figure 8A shows that the specific CA inhibitors ethoxyzolamide (EZA) and acetazolamide (AZA) diminish the accelerating action of glucose. The resting beat frequency of 3.29±0.17 Hz (EZA) and 3.31±0.20 Hz (AZA) increases within 10 min to 4.69±0.09 Hz (glucose + EZA) and 4.62±0.09 Hz (glucose + AZA). These values are significantly diminished compared to those of the control measurements (6.42±0.11 Hz) in which cells were simply stimulated with glucose over 30 min. During removal of EZA, beat frequency rises to 6.28±0.14 Hz and to 6.31±0.15 Hz after withdrawal of AZA.

If sAC is involved in the accelerating action of glucose, the inhibition of this enzyme should prevent an increase in sperm beat frequency. Figure 8B shows that in the simultaneous presence of 2'OH-E and glucose, the basal beat of 3.23±0.13 Hz increases within 10 min to 4.61±0.20 Hz and to 6.18±0.11 Hz after removal of the inhibitor. However, the application of glucose without 2'OH-E leads to an increase of the beat from 3.24±0.15 Hz to 6.10±0.17 Hz during the first 10 min. A similar effect is seen when applying KH7 (Fig. 8C). Here, the resting beat frequency accelerates within 10 min from 3.52±0.23 Hz to 4.27±0.23 Hz (KH7+ glucose) and to 6.16±0.15 Hz (glucose), while in the control measurements, in which cells were stimulated merely with glucose over 40 min, the beat frequency changes during 10 min from 3.37±0.21 Hz to 5.98±0.18 Hz.

If cAMP-mediated pathways are a partial determinant of the accelerating action of glucose, then a blockade of the PKA target of cAMP action should block acceleration. Figure 8D shows that PKA inhibitor H89 slows the acceleration by glucose resulting in 4.80±0.11 Hz after 10 min, whereas in the control measurements, sperm beat rises to 6.42±0.11 Hz during the same period of time. Taken together, the results presented suggest that the accelerating action of glucose involves cAMP-mediated pathways as well as the production of HCO_3_−.

## Discussion

Lactate, pyruvate and glucose are components of female genital tract fluids and are used by spermatozoa as glycolytic and respiratory energy substrates. It is known not only that sperm alkalinize physiologically during maturation in the female genital tract but also that glycolytic key enzymes show greater activity at alkaline pH levels. We therefore examined to what extent the pH_i_ of sperm influences flagellar beat in dependence on glycolysis.

Glycolysis is the main mechanism for murine sperm to produce energy in terms of ATP [2], [3], [4]. However, it is unclear if glycolysis is affected during changes in pH_i_ as it occurs during sperm maturation in the female genital tract [26], [27]. We show that sperm beat frequency averaged at ∼3.5 Hz as long as lactate or pyruvate were present in the external medium, irrespective of the co-application of glucose. However, in the sole presence of glucose, beat frequency nearly doubled within 10 min and remained stable for at least 60 min, however declined to basal values upon reapplication of lactate or pyruvate. We have previously shown by means of photometry that sperm exhibit monocarboxylate/H+ co-transporter activity resulting in changes in pH_i_ [2]. With this work, we present data that show a significant rise in sperm beat frequency when both the proton and the lactate anion gradients are expanded. On the basis of these data we propose that MCTs exhibit greater activity under such conditions as demonstrated for astrocytes [28] and that pH_i_ is involved in the accelerating effect of glucose. The pH_i_ of sperm incubated in buffer containing glucose exhibited a pH_i_ of ∼7.1 which decreased during application of pyruvate and lactate. To determine whether the elevation of beat frequency was mediated by glycolysis or solely by changing the pH_i_, we stimulated sperm with the non-glycolyzable substrate 2DG, as well as with the weak base MA and the weak acid propionate. 2DG is a structural glucose analog which is taken up by glucose transporters (GLUT) [29], of which GLUT8, GLUT9a and GLUT9b have been shown to localize to the acrosome and the tail of murine sperm [30], [31], [32]. Indeed, 2DG is phosphorylated by hexokinase to 2DG-phosphate but it cannot be further metabolized by glucose-6-phosphate dehydrogenase or phosphohexoisomerase, thus inhibiting glycolysis [33], [34]. In the presence of 2DG, basal sperm beat frequency of ∼3.5 Hz declined significantly after 10 min which is in agreement with data of Mukai and Okuno [35]. However, subsequent application of glucose restored this effect, resulting in a doubling of the initial beat frequency. Although MA evoked an alkaline shift and propionate induced an acidic shift of pH_i_ the resting beat of ∼3.5 Hz remained unchanged. Taken together, these results primarily indicate that acceleration of sperm beat frequency requires glycolysis and is not merely evoked by changes in pH_i_.

Bock and others showed that the activity of the glycolytic key enzyme phosphofructokinase is greater under alkaline conditions [3], [11], [26]. For the rabbit muscle, it was demonstrated that this enzyme dissociates into inactive subunits under acidic conditions and re-associates into the active form when the pH is raised again [6], [7], [36]. In quiescent epididymal mouse spermatozoa hexokinase activity was significantly lower compared to that of sperm incubated in physiological buffer [36]. To investigate whether the accelerating effect of glucose can be modulated by a more alkaline or acidic pH_i_, we stimulated sperm with buffer containing glucose and MA, as well as glucose and propionate. Co-application of glucose and MA lead to an alkalinization within 70 s, whereas co-application of glucose and propionate lead to an acidification within 50 s. The results showed that sperm beat frequency did not increase in the concomitant presence of glucose and propionate. However, the addition of glucose and MA lead to an increase of beat frequency which was more pronounced after 5 min than that observed in the presence of glucose only. To further connect the pH-dependent acceleration of flagellar beat with glycolysis, we also determined the intracellular ATP content of sperm incubated with varying energy sources. Over a time period of 30 min, the ATP content decreased by about 50% in the presence of 2DG. This is not surprising since it is known that accumulation of 2DG-phosphate leads to depletion of the intracellular ATP content [33], [34], [37]. Supplying sperm with lactate resulted in a constantly maintained ATP level, whereas feeding sperm with glucose produced a doubling of the ATP content. Co-application of glucose and MA further increased the amount of ATP, whereas stimulation with lactate and MA did not change the ATP content. We also investigated the absorption of NADH/NADPH since both glycolysis and the citrate cycle yield such reduction equivalents. With glucose and lactate present, absorption values are reduced by about nearly 8% compared to the values obtained when only glucose is present in the buffer. When applying 2DG instead of glucose, absorption values are reduced by about nearly 20%. This results from the fact that 2DG can only be phosphorylated by hexokinase but cannot be further processed by subsequent glycolytic enzymes. Similar low values were apparent when applying lactate only. On the basis of these results we propose, on the one hand, that the rate of glycolysis is favourably influenced by a more alkaline pH_i_. This is in accordance with the fact that glycolytic key enzyme activity shows pH dependency as aforementioned [3], [11], [26], as demonstrated in leukocytes [9] and erythrocytes [10]. On the other hand, we assume that murine sperm are unable to accelerate flagellar beat with lactate for two reasons: (i) lactate import follows the same mechanisms as pyruvate transport. It is also accompanied by a decrease in pH_i_, thus slowing import into mitochondria where conversion into pyruvate by the sperm specific lactate dehydrogenase C (LDH C) takes place. (ii) LDH C is assumed to be located in the cytoplasm [38] and in the mitochondrial matrix [38], [39] to catalyze lactate oxidation under aerobic conditions. Nevertheless, pyruvate generated in the cytoplasm would not be transported into mitochondria due to the low pH_i_. Although we did not measure the overall activity of glycolytic key enzymes, the results presented in this section suggest that a more alkaline pH_i_ supports the glycolytic rate in spermatozoa, whereas a more acidic intracellular milieu does not favor the glycolysis-dependent acceleration of the flagellar beat and ATP production. Possible channels contributing to intracellular alkalinization in murine spermatozoa are monocarboxylate/H+ co-transporters [2] and sperm specific Na+/H+ exchangers (sNHE) [40]. It is unlikely that the recently identified major proton channel Hv1 of human sperm supports intracellular alkalinization in murine sperm since Hv1 activity in murine cells exhibits only a fractional amount of the human sperm mean outward current density of ∼4% [41].

Since glycolytic end products are mainly translocated into mitochondria to feed the citric acid cycle, we confirmed that the glucose-mediated acceleration of flagellar beat requires mitochondria and applied DCA and AOA in the presence of glucose. DCA is an inhibitor of the pyruvate dehydrogenase kinase, thus preventing mitochondria to oxidize pyruvate to acetyl-coA [42]. AOA, in general, inhibits pyridoxal phosphate-dependent enzymes, such as aspartate aminotransferase, which is a component of the malate-aspartate shuttle. Once inhibited, cytosolic NADH no longer can be re-oxidized, thus leading to a breakdown of the respiratory chain [43]. In the presence of DCA or AOA, sperm beat frequency averaged at basal levels of ∼3 Hz, whereas washing out of the drugs led to a doubling of flagellar beat during 30 min in the sole presence of glucose. We also applied succinate - a direct substrate of the citric acid cycle - to sperm to further confirm that acceleration of flagellar beat requires respiratory substrates. Succinate import into cells is driven by Na+-dependent dicarboxylate transporters (NaDCs) which belong to the SLC13 gene family [44], [45]. Murine orthologues of NaDC1 (SLC13A2) and NaDC3 (SLC13A3) are expressed in a variety of tissues, such as kidney [46], [47], [48] testis (http://mrg.genetics.washington.edu) and caput epididymidis [49]. However, the presence and activity of these proteins in sperm has not yet been determined. In contrast to MCTs, NaDC activity is mainly electroneutral, which is consistent with our observation of the slight pH_i_ shift during the application of succinate. Succinate import into mitochondria is mediated in many organs, such as liver and kidney, by the dicarboxylate carrier SLC25A10, which catalyzes the electroneutral exchange of succinate and inorganic phosphate [50]. Expression of SLC25A10 has been demonstrated to occur in the testis (http://mrg.genetics.washington.edu), but protein distribution remains unknown as yet. Since we observed a doubling of beat frequency within 10 min from ∼3.5 Hz to ∼6 Hz, we propose that succinate transporters are functionally active in sperm. However, during the stimulation with pyruvate - the end product of aerobic glycolysis - sperm beat frequency remained unchanged over 20 min. Pyruvate transport into mitochondria by the pyruvate carrier [51], [52] is accompanied by a stoichiometric proton flux [53] and driven by the trans-membrane H+ gradient [51], [54]. Studies with isolated rat liver mitochondria preloaded with [2–14C] pyruvate showed that substrate efflux across the mitochondrial membrane increased significantly at alkaline pH compared to neutral or acidic pH [55]. From these results we conclude not only that the mitochondrial pyruvate carrier is active in both directions but also that the pyruvate import rate changes with altering pH_i_. Since the addition of pyruvate to the external medium leads to a co-import of protons, we propose that intracellular acidification reduces pyruvate uptake into mitochondria. To test this hypothesis, we supplied sperm with pyruvate and MA to diminish the decrease of pH_i_. During co-application of both substrates, sperm beat increased significantly within 10 min from ∼3.0 Hz to ∼4.5 Hz. Since sperm do not fully accelerate flagellar beat as they do in the presence of glucose, we suggest that the pH_i_ modulates the activity of the mitochondrial pyruvate carrier and that the glucose-mediated increase of flagellar beat requires mitochondria.

However, the generation of energy in terms of ATP does not explain the doubling of beat frequency, since, so far, only HCO_3_− is known to triple the beat within 20 s [25]. We have evidence that sperm exhibit carbonic anhydrase (CA) II activity (unpublished data) which reversibly catalyzes hydration of CO_2_ to HCO_3_− and protons. Since cell respiration yields CO_2_, we assumed that the glucose-mediated rise in sperm beat frequency cannot only be explained by the production of ATP but also by the generation of HCO_3_−. Downstream pathways of HCO_3_− include direct activation of the sperm-specific soluble adenylyl cylase (sAC) [12], [13], elevation of the intracellular cAMP content and protein phosphorylation of several target proteins by protein kinase A (PKA) [14], [15]. We therefore investigated the glucose-mediated acceleration of flagellar beat during inhibition of CAs by EZA and AZA, sAC by KH7 and 2'OH-E and PKA by H89. These chemicals have been shown to successfully block the response to HCO_3_− and capacitation-associated steps [24], [25], [56], [57]. In these experiments, basal beat frequency (3.5 Hz) rose slightly during inhibitory conditions. However, during removal of the respective inhibitor, flagellar beat increased significantly to nearly double in the sole presence of glucose. Since each inhibitor was applied in a concentration that allowed a quick recovery after removal, we therefore interpret the slight rise of beat frequency as an incomplete inhibitory effect. The experiments were conducted under normoxia, which does not fully represent the hypoxic milieu of the female genital tract. However, we have previously shown that the application of 5% CO_2_ to the perifusion leads to a sustained increase of sperm beat frequency within 40 s. This results from CA IV activity [24] and hinders any examination of the glucose-mediated rise in beat frequency under more physiological conditions. Although we neither showed an increase of intracellular CO_2_ nor the metabolization to HCO_3_−, our results suggest that cAMP-mediated pathways that are promoted by the stimulatory action of HCO_3_− on sAC, are connected to glycolysis.

From the results presented, we propose the following model (Fig. 1C): as long as lactate or pyruvate are present in the external medium, such as in the vaginal fluid, pH_i_ is maintained at <7.0 due to the electroneutral co-transport of monocarboxylate anions and protons. When sperm enter a medium that is rich in glucose but poor in lactate/pyruvate, such as the uterine fluid, glucose will be taken up by GLUT transporters and protons will leave the cell, thus leading to an intracellular alkalinization to pH_i_ values of >7.0. Alkalinization, in turn, will promote glycolysis, yielding more pyruvate and - due to its metabolism during the mitochondrial tricarboxylic acid (TCA) cycle - a surplus of CO_2_. A portion of CO_2_ will then be hydrated to HCO_3_− by intracellular CAs thus initiating downstream effects which result in an increase of beat frequency.

In summary, we suggest that glucose consumption not only yields ATP but also is connected to pH-dependent generation of HCO_3_−. We support the idea that these interrelated events can be seen as an additive mechanism to enhance flagellar beat, thereby contributing to fertility. Furthermore, we propose that changes in substrate composition of vaginal, uterine and fallopian tube fluid induce changes in sperm pH_i_, thereby regulating the generation of HCO_3_− in the respective part of the female genital tract.

## Supporting Information

Figure S1 **Both the proton and the lactate anion gradient are driving forces to contribute to the glucose-mediated enhancing effect on sperm beat frequency.** Sperm were initially perifused in all three experiments with buffer HS containing 10 mM lactate and 5 mM glucose with a pH of 7.4. Cells were then continuously perifused with buffer HS containing 5 mM glucose, less lactate (5 mM) and less protons (pH 8.0) for 30 min, before switching to buffer HS (pH 8.0) containing 5 mM glucose only (solid black line). For control measurements, sperm were either stimulated over 50 min with buffer HS (pH 8.0) containing 5 mM glucose and 5 mM lactate (dotted black line) or bathed for 50 min in buffer HS (pH 8.0) with 5 mM glucose only (gray line). Significant changes were analyzed relating to t  = 0 min and averaged at **p<0.05 (t ≥25 min) and at ***p<0.001 (t ≥45 min) under reduced lactate and proton concentration. In the control, significance values of ***p<0.001 at t ≥15 min were manifest. Shown are mean values±s.e. of 30 cells of 3 animals.
(TIF)
