# Supplementary figures and images for: Glucose is a pH-Dependent Motor for Sperm Beat Frequency during Early Activation
Source: PLoS One. 2012 Jul 20;7(7):e41030. doi: 10.1371/journal.pone.0041030 (PMC3401232; doi:10.1371/journal.pone.0041030)

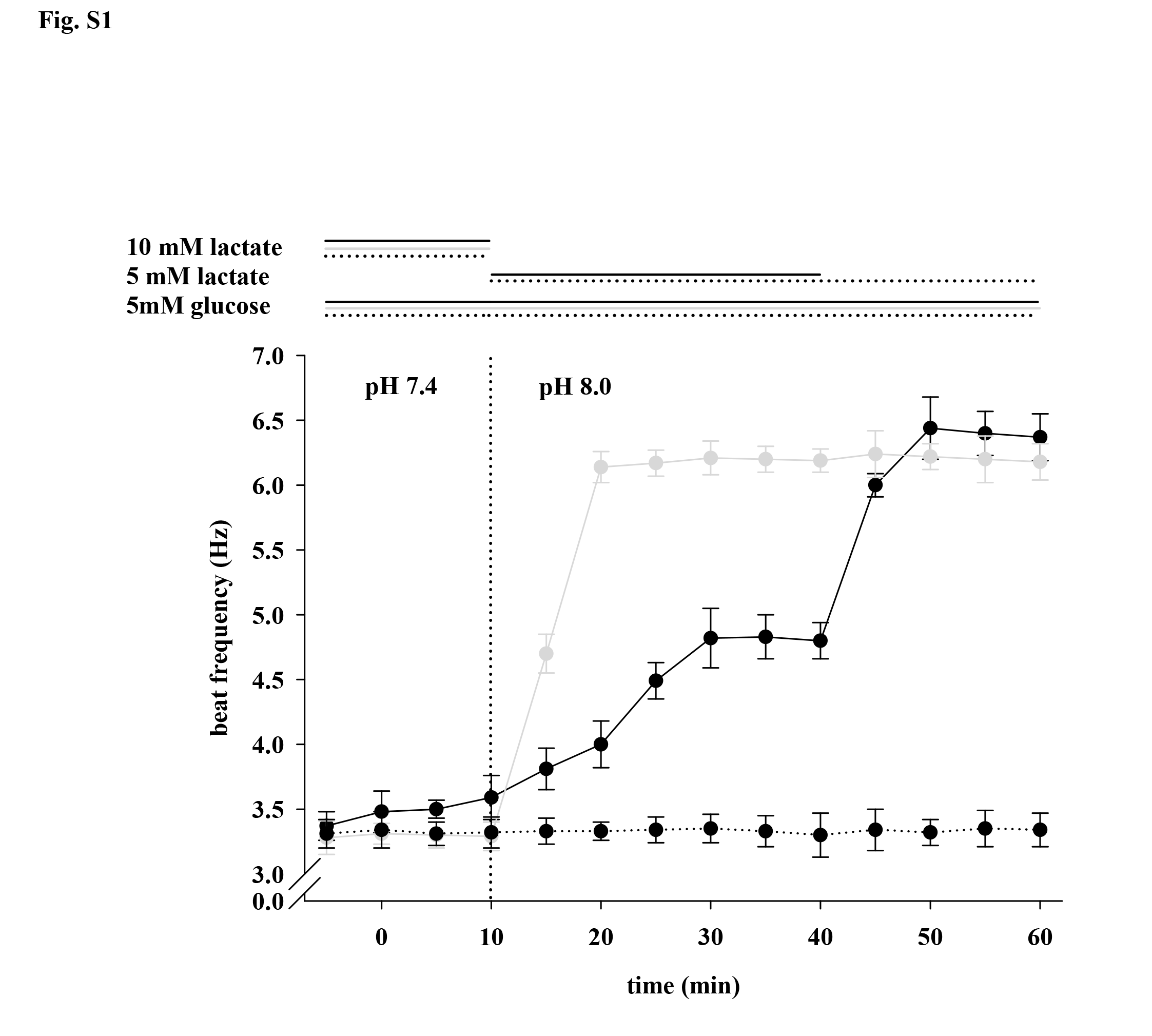

Supplement: Figure S1 — Both the proton and the lactate anion gradient are driving forces to contribute to the glucose-mediated enhancing effect on sperm beat frequency. Sperm were initially perifused in all three experiments with buffer HS containing 10 mM lactate and 5 mM glucose with a pH of 7.4. Cells were then continuously perifused with buffer HS containing 5 mM glucose, less lactate (5 mM) and less protons (pH 8.0) for 30 min, before switching to buffer HS (pH 8.0) containing 5 mM glucose only (solid black line). For control measurements, sperm were either stimulated over 50 min with buffer HS (pH 8.0) containing 5 mM glucose and 5 mM lactate (dotted black line) or bathed for 50 min in buffer HS (pH 8.0) with 5 mM glucose only (gray line). Significant changes were analyzed relating to t = 0 min and averaged at **p<0.05 (t ≥25 min) and at ***p<0.001 (t ≥45 min) under reduced lactate and proton concentration. In the control, significance values of ***p<0.001 at t ≥15 min were manifest. Shown are mean values±s.e. of 30 cells of 3 animals. (TIF) [file pone.0041030.s001.tif]
